# Interaction of RNA polymerase II and the small RNA machinery affects heterochromatic silencing in Drosophila

**DOI:** 10.1186/1756-8935-2-15

**Published:** 2009-11-16

**Authors:** Harsh H Kavi, James A Birchler

**Affiliations:** 1 Division of Biological Sciences, University of Missouri, Columbia MO-65211, USA

## Abstract

**Background:**

Heterochromatin is the tightly packaged dynamic region of the eukaryotic chromosome that plays a vital role in cellular processes such as mitosis and meiotic recombination. Recent experiments in *Schizosaccharomyces pombe *have revealed the structure of centromeric heterochromatin is affected in RNAi pathway mutants. It has also been shown in fission yeast that the heterochromatin barrier is traversed by RNA Pol II and that the passage of RNA Pol II through heterochromatin is important for heterochromatin structure. Thus, an intricate interaction between the RNAi machinery and RNA Pol II affects heterochromatin structure. However, the role of the RNAi machinery and RNA Pol II on the metazoan heterochromatin landscape is not known. This study analyses the interaction of the small RNA machinery and RNA Pol II on *Drosophila *heterochromatin structure.

**Results:**

The results in this paper show genetic and biochemical interaction between RNA Pol II (largest and second largest subunit) and small RNA silencing machinery components (*dcr-2, ago1, ago2, piwi, Lip [D], aub *and *hls*). Immunofluorescence analysis of polytene chromosomes from trans-heterozygotes of RNA Pol II and different mutations of the small RNA pathways show decreased H3K9me2 and mislocalization of Heterochromatin protein-1. A genetic analysis performed on these mutants showed a strong suppression of *white-mottled4h *position effect variegation. This was further corroborated by a western blot analysis and chromatin immunoprecipitation, which showed decreased H3K9me2 in trans-heterozygote mutants compared to wild type or single heterozygotes. Co-immunoprecipitation performed using *Drosophila *embryo extracts showed the RNA Pol II largest subunit interacting with Dcr-2 and dAGO1. Co-localization performed on polytene chromosomes showed RNA Pol II and dAGO1 overlapping at some sites.

**Conclusion:**

Our experiments show a genetic and biochemical interaction between RNA Pol II (largest and second largest subunits) and the small RNA silencing machinery in *Drosophila*. The interaction has functional aspects in terms of determining H3K9me2 and HP-1 deposition at the chromocentric heterochromatin. Thus, RNA Pol II has an important role in establishing heterochromatin structure in *Drosophila*.

## Background

The metazoan chromosome consists of two distinct functional compartments based mainly on their transcriptional competence and higher order chromatin packaging. Heterochromatin is tightly packed and has a paucity of actively transcribed genes. It plays a vital role in biological functions such as determining the distribution of meiotic recombination, telomere maintenance and sister chromatid cohesion [1-3]. The metazoan chromosome is also interspersed with facultative heterochromatin, which has the potential to become transcriptionally competent. This fine tuning ensures gene regulation in a cell specific and spatio-temporal manner during development.

The long held notion that heterochromatin is refractory to transcription was reversed in recent experiments performed in *Schizosaccharomyces pombe *and mouse cells [4,5]. It was demonstrated that centromeric heterochromatic repeats are transcribed in the late S phase of the cell cycle and this transcription of heterochromatic repeats is essential for the structural maintenance of centromeric heterochromatin. It was also shown that heterochromatin is a versatile platform with proteins such as SWI6, which prevents access of RNA Polymerase II to centromeric repeats, in dynamic equilibrium with Epe1, which promotes transcription [6]. During transcription through the heterochromatic arrays, H3S10phos increases and SWI6 deposition is decreased, thus decondensing the heterochromatin structure. During the late S phase, transcription of heterochromatic repeats by RNA Pol II occurs and an increase in the deposition of Ago1, Clr4 and Rik1 is also observed [4]. The accumulation of Clr4 histone methyl transferase, together with the RNAi induced transcriptional silencing (RITS) complex components, results in the processing of cen siRNAs, which would then direct the methylation of H3K9 at heterochromatic repeats. It was also shown in *S. pombe *that transcription of the centromeric repeats produces nascent transcripts, which are used as a template by the RNA-dependent RNA polymerase complex (RDRC) to produce dsRNA. The latter is then cleaved by Dicer to synthesize centromeric siRNA which are then loaded onto RITS, bringing about the deposition of H3K9me2 (Clr4 mediated) and SWI6 at the centromeric heterochromatin [7-10]. It was also shown that in fission yeast mutations in the second largest and fourth largest subunit of RNA polymerase II affects the synthesis of centromeric and pre-centromeric siRNAs, respectively [11,12] and was accompanied by reduction of H3K9me2 and Swi6 at the centromeres. The mutations in RNA Pol II subunits did not cause any significant changes in global transcription but its effect was confined to the centromeric heterochromatin structure. These studies highlighted the fact that RNA Pol II performs an integral function for centromeric heterochromatin structural maintenance in conjunction with the small RNA processing machinery.

We explored the role of RNA Polymerase II on heterochromatin structure in *Drosophila *because of the availability of polymerase mutations and a well-developed model system for the study of heterochromatin. We employed genetic, biochemical and cytological analysis to address this issue. Our analysis indicates that the largest and second largest subunits of RNA polymerase II interact genetically and biophysically with RNA silencing machinery components. Our data also indicate that RNA Pol II mutants (largest and second largest subunits) link transcription and RNA silencing components to heterochromatin structure in metazoans.

## Results and discussion

**Figure 1:**
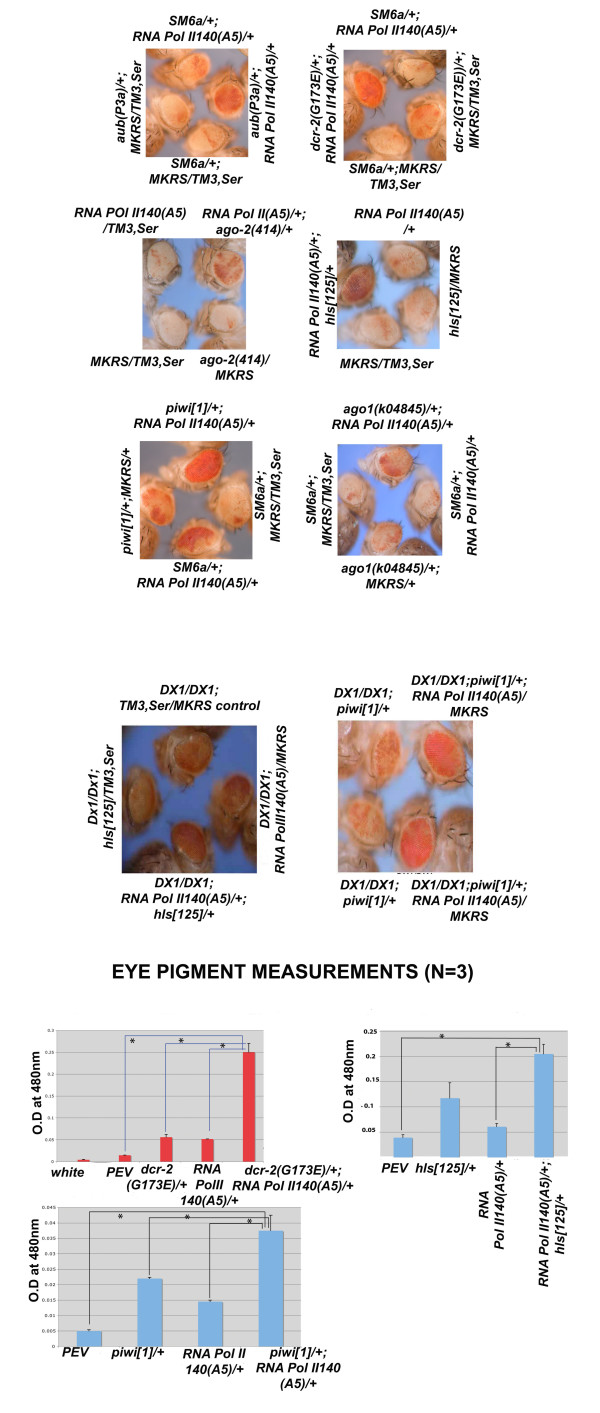
**Suppression of heterochromatic silencing in trans heterozygotes of RNA Pol II 140 and RNA silencing machinery mutants**. The genotypes of control, single heterozygous and trans-heterozygous male flies are indicated involving the X chromosome inversion *In(1)w [m4h]*. The effect on DX1 tandem *mini-white *silencing is also shown in the middle. The genotypes of control, single heterozygous and trans-heterozygous male flies are indicated. The alellic combinations used are noted. Measurement of eye pigment analysis is presented below. Measurements from three independent experiments are shown. Error bars represents standard error. Optical density values at 480 nm are indicated on the Y-axis and the genotypes represented on the X-axis. Asterisks indicate significant *P *values < 0.05.

In order to test the role of RNA Pol II on heterochromatin, we employed genetic tests using the inversion of white-mottled4h stock. The *In(1)w [m4h] *stock has a pericentric inversion between the white gene and the centric heterochromatin. This arrangement results in a variegated eye pattern. Many genes acting as chromatin modifiers suppress or enhance the position-effect variegation (PEV) effect. We used mutations in the second largest subunit of RNA Pol II 140. The mutant alleles used were *RNA Pol II 140 (A5) *and *RNA Pol II 140 (wimp)*. The A5 allele is a null mutant with a five amino acid deletion while *wimp *is an antimorph [13,14]. We observed that RNA Pol II mutations weakly suppressed PEV as a heterozygote. However, the trans-heterozygote of *RNA Pol II 140(A5) *and dicer-2 (*dcr-2 G173E*) showed a stronger suppression of PEV when compared with either the single heterozygotes or control normal male flies (Figure 1A and 1B). This experiment revealed genetic interaction between RNA Pol II and Dicer-2, which is a central processing enzyme in the RNAi pathway.

To further understand the nature of the interactions between RNA Pol II and the RNA silencing machinery, we tested the effect of an array of different RNA silencing machinery mutations on PEV including those implicated in piRNA formation, which acts independently of Dicer [15]. In each case, different mutant alleles of each gene were tested in order to rule out any linked gene effect on PEV suppression. The PEV analysis indicated genetic interactions between *dcr-2(L811fsX), dcr-2(G173E), ago-2(414), piwi[1], piwi[2], hls [616], hls [125], aub [QC42 ], aub [P3a] *and RNA Pol II alleles (Figure 1A and 1B and Additional file 1). In each case the trans heterozygotes of RNA Pol II and RNA silencing pathway mutations exhibited stronger suppression of PEV than single heterozygotes. However, the suppression of PEV was strongest in the trans-heterozygotes of RNA Pol II and *dcr-2 *alleles among all the other combinations tested as indicated by the eye pigment measurements.

The suppression of PEV is a reflection of the changes in the chromatin structure of heterochromatin. The effect of RNA Pol II and RNA silencing machinery trans-heterozygote mutants was not confined to the heterochromatin environment of the chromocentre. This was shown in an experiment employing transgenic flies that have seven tandem copies of *mini-white*, referred to as DX1 (Figure 1C), that are located in the euchromatin of chromosome 2. Flies that are homozygous for this transgene arrangement have a heterochromatin environment around the *mini-white *arrays, thus silencing the expression of *mini-white *transgenes in mosaic fashion [16]. We tested two combinations, namely RNA *Pol II 140(A5) *and *piwi[1]*, as well as *RNA Pol II 140 (A5) *and *hls [125] *on DX1 homozygous flies. In each case the trans-heterozygotes reversed the silencing of *mini-white *to a much greater extent when compared to either single heterozygotes or control flies with no mutations. The exact molecular mechanism of DX1 silencing has not been elucidated but is believed that pairing sensitive silencing might be one of the contributing factors. The experiments performed with RNA Pol II mutants suggest that there is also involvement of a transcriptional silencing component.

**Figure 2:**
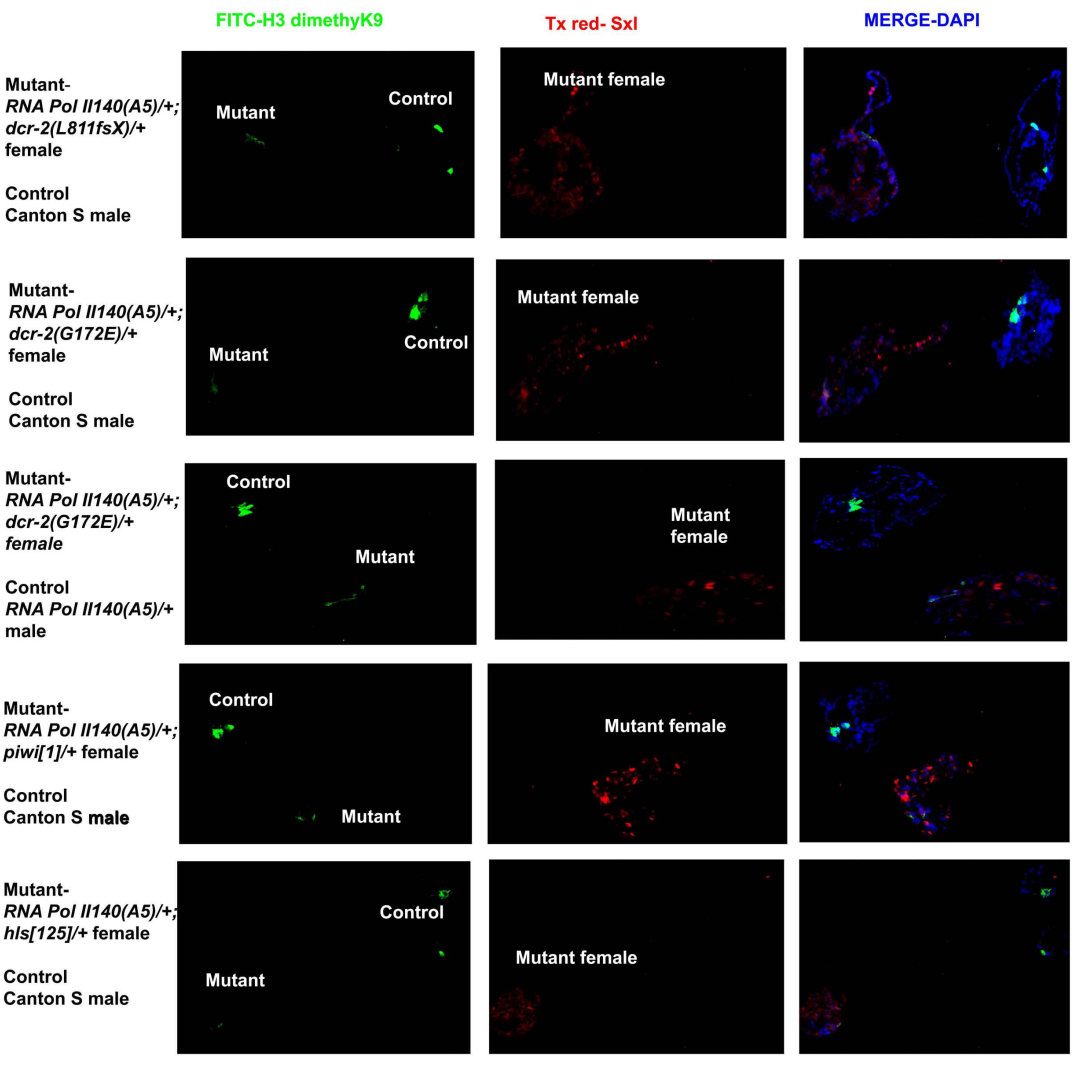
**Immunofluorescence analysis of polytene chromosomes in RNA Pol II 140 and small RNA pathway mutants**. Representative images from five different experiments (approx 50 pairs of nuclei) have been examined. The genotypes of each polytene nuclei have been indicated. The FITC (green) channel shows H3K9me2 antibody signal while the Texas Red shows Sxl antibody signal. H3K9me2 staining at the chromocentre is displayed.

The strong suppression of PEV in trans-heterozygotes of RNA Pol II and RNA silencing machinery components led us to investigate the heterochromatin structure at the chromocentre of polytene chromosomes. In *Drosophila*, H3K9me2 modification is concentrated at the centric heterochromatin. H3K9me2 is also interspersed along the euchromatin arms where it is accumulated on transposable elements [17]. We reasoned that, because the suppression of the *white *gene is relieved in the PEV analysis, H3K9me2 at the chromocentre would be reduced. We performed experiments using third instar larval polytene chromosomes probed with antibodies against H3K9me2. We combined the trans-heterozygotes and the control wild type chromosomes in the same preparation so that they could be observed in one microscopic field for direct comparisons under identical experimental conditions. As our PEV analysis indicated that the trans-heterozygote of *RNA Pol II 140 (A5) *and *dcr-2 (G173E) *suppressed PEV very strongly, we analysed this combination for reduction in H3K9me2 at the chromocentre of polytene chromosomes. Indeed, compared to the wild type nuclei, *RNA Pol II 140 (A5)/+; dcr-2 (G173E)/+ *showed a reduction of H3K9me2 as visualized by the immuno-fluorescence experiments (Figure 2). A similar pattern showing decreased H3K9me2 deposition at the chromocentre was observed using the *RNA Pol II 140(wimp)/+; dcr-2 (G173E)/+ *combination and *RNA Pol II 140(A5)/+; dcr-2 (L811fsX)/+*, which illustrates the generality with regard to different alleles at both loci. We then performed immunofluoresence experiments on polytene chromosomes using *RNA Pol II 140 (A5)/*+ as a control. In accordance with our PEV analysis, *RNA Pol II 140 (A5)/+; dcr-2 (G173E)/+ *showed decreased H3K9me2 deposition at the chromocentre compared with the single heterozygote of *RNA Pol II (A5)/+*. The experiments were repeated five times with about 75 pairs of mutant and control nuclei observed. In each case about 75%-80% of the mutant nuclei showed a reduction of H3K9me2 at the chromocentre compared to the wild type. All the experiments were performed by switching the sexes of mutant and normal using antibodies against Sex-lethal, which is only expressed in females, to distinguish male from female nuclei. This was done in order to ensure that the reduction in H3K9me2 at the chromocentre was not sex specific. We then analysed polytene chromosomes using wild type control and trans-heterozygotes of: (1) *RNA Pol II 140 (A5)/+; hls [125]/+*; (2)*RNA Pol II 140(A5)/+; hls [E61]6/+*; (3)*RNA Pol II140 (A5)/+; piwi[1]/+*; and (4)*RNA Pol II 140(A5)/+; Lip [D]/+ *[18] (Figure 2 and Additional file 2). *Lip *is synonymous with Dmp68 [19], which have been shown to be necessary for RNAi in tissue culture cells [20]. In each case the trans-heterozygote mutants showed reduced H3K9me2 deposition at the chromocentre. The immunofluoresence analysis of polytene chromosomes using trans-heterozygotes complements the PEV phenotypic analysis. These experiments indicate that RNA Pol II exhibits genetic interaction with the RNA silencing machinery components and that the suppression of PEV is correlated with a reduction in H3K9me2 at the chromocentre.

**Figure 3:**
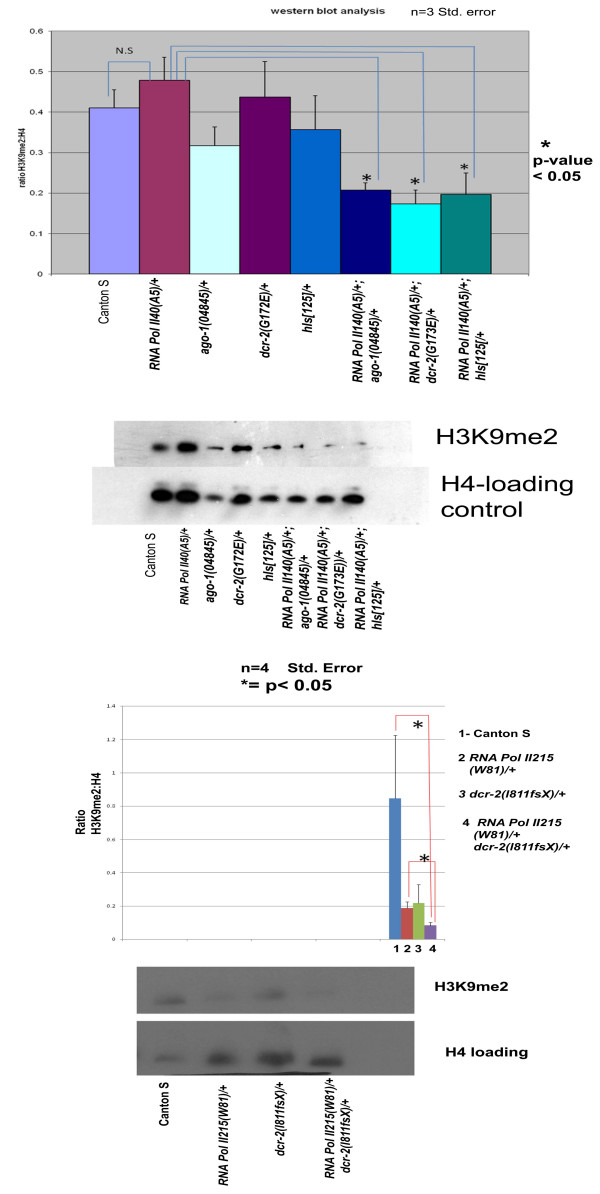
**Western blot analysis of H3K9me2 levels in RNA Pol II 140 and small RNA pathway mutants**. Adult carcasses were used for acid extraction of histones. Results from three independent biological replicates are shown. Western blot analysis of acid extracted histones from adult carcasses of indicated genotypes is shown. Asterisk shows p value < 0.05. Standard error from four different experiments is shown.

In order to quantify the reduction of H3K9me2, western blot analysis was performed on acid extracted histones using H3K9me2 antibodies. Adult carcasses, with the gonads removed, were used to rule out any effect of the RNA silencing machinery in the germline [21]. The analysis revealed that single heterozygotes of *RNA Pol II 140(A5)/+ *and RNA silencing machinery mutants alone showed very modest to no change in H3K9me2 levels compared to the wild type. However, trans-heterozygotes for the combined mutants showed a strong reduction in H3K9me2 levels compared with wild type and single heterozygotes (Figure 3). The western blot analysis also corroborated the PEV analysis.

**Figure 4:**
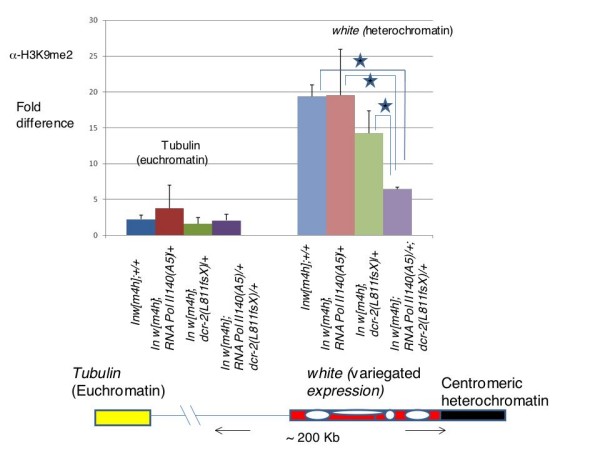
**Chromatin Immunoprecipitation analysis of H3K9me2 levels in the RNA Pol II 140 and small RNA pathway mutants**. Chromatin immunoprecipitation analysis of indicated genotypes on the X axis was performed. The Y axis indicates fold difference changes in H3K9me2 at the *white* and *tubulin* loci between different genotypes. The figure at the bottom shows the inverted arrangement of the *white* locus in the vicinity of centromeric hetrochromatin and is enriched in H3K9me2. The tubulin locus is in euchromatin and is not significantly enriched in H3K9me2. Asterisk indicates P < 0.05 from four different replicas.

We next performed chromatin immunoprecipitation (ChIP) using H3K9me2 antibodies on adult flies. The combination of RNA Pol II and dcr-2 was selected because it gave the strongest suppression of PEV in the *w [m4h] *background. The ChIP analysis revealed significant enrichment of H3K9me2 at the *white *locus in the vicinity of centromeric heterochromatin (*w [m4h] *genetic background). The tubulin locus (in euchromatin) did not show any enrichment of H3K9me2 nor any significant difference in the amount of H3K9me2 between the control (*In(1)w [m4h];+/+*), single heterozygous mutants and the double heterozygotes of RNA Pol II 140 and *dcr-2(L811fsX*). However, at the *white *(which lies in the vicinity of centromeric heterochromatin due to the inversion) locus, the double heterozygotes of *RNA Pol II 140 (A5)/+; dcr-2 (L811fsX) *showed significant reduction (about fourfold) of H3K9me2 compared to the control as well as to single heterozygote mutants (Figure 4). Also, there was no significant change in H3K9me2 between the control and single heterozygotes of *RNA Pol II 140 *and *dcr-2*. The ChIP analysis at the *white *locus in the *In(1)w [m4h] *genetic background indicates the importance of H3K9me2 in suppressing the *white *locus. The ChIP results are consistent with the PEV analysis and Western blot results.

In addition to the second largest subunit of RNA Pol II, we also studied the effect of the RNA Pol II's largest subunit mutation on H3K9me2 levels in the adult carcass (Figure 3). As the largest subunit gene (*RNA Pol II215 W81*) is located on the X-chromosome, a PEV analysis of male flies was not possible and the fact that a translocation balancer chromosome between the X chromosome and the second chromosome was not available precluded any immunofluoresence analysis on larval polytene nuclei. The mutant allele used was W81, which has a truncated carboxyl terminal domain (CTD) due to the presence of a premature stop codon.

Trans-heterozygotes of *RNA Pol II215(W81)/+; dcr-2(L811fsX) *showed a significant reduction of H3K9me2 in Western blot analysis compared to the wild type as well as *RNA Pol II215(W81)/+ *alone. The reduction of H3K9me2 with two different subunits of RNA Pol II, in combination with *dicer-2 *mutations, provides further evidence of a role of RNA pol II in heterochromatin formation in conjunction with RNA silencing genes.

**Figure 5:**
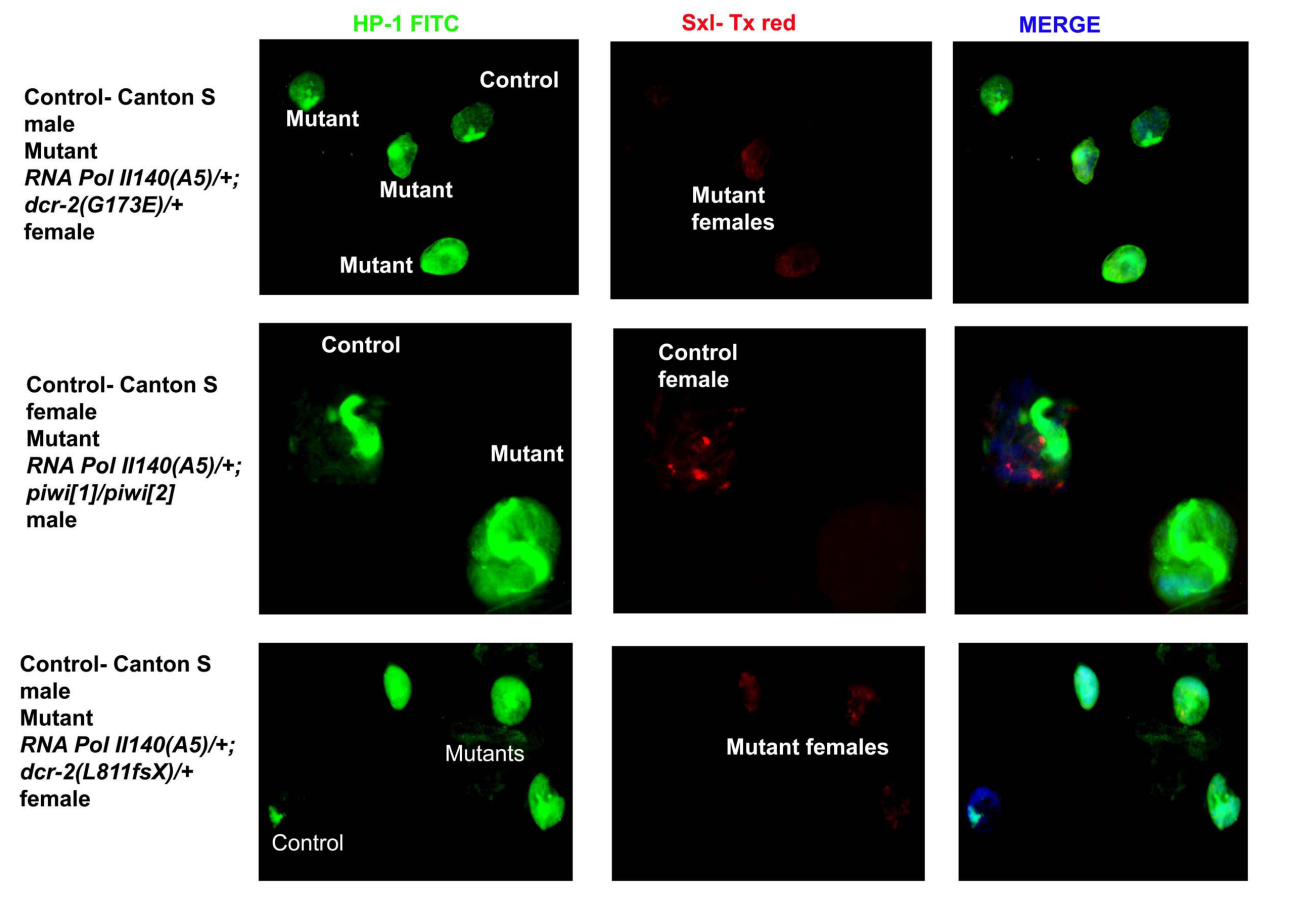
**Heterochromatin protein-1 (HP1) mislocalization in RNA Pol II and small RNA pathway mutants**. HP1 mislocalization is visualized in gently squashed polytene nuclei of the noted genotypes. HP1 (FITC) is shown in green and Sxl (Tx red) is shown in red.

The chromocentre of *Drosophila *is characterized by strong deposition of heterochromatin protein-1 (HP1). With a reduction of H3K9me2, HP1 is deposited at various low affinity-binding sites along the chromosome arms [22]. The presence of H3K9me2 provides high affinity binding sites for the docking of HP1. We examined the polytene chromosomes of RNA *Pol II 140 (A5)/+; dcr-2(G173E) *trans-heterozygotes for any changes in HP1 deposition pattern (Figure 5). The gently squashed polytene nuclei from third instar larvae showed mislocalization of HP1 to the euchromatin arms compared to wild type nuclei, which showed a much more discrete HP1 deposition at the chromocentre.

With regard to piRNA genes, previous experiments involved examining HP1 mislocalization in *piwi*[1]/piwi[2] heteroallelic mutants. This combination did not show a major mislocalization of HP1 [23]. To test the impact of RNA pol II, we introduced the *RNA Pol II140(A5)/+ *mutation in this background. This combination caused an obvious mislocalization of HP1 (Figure 5).

To establish whether the cytological observations represented a mislocalization or a quantitative difference, we used a Western blot analysis which indicated that HP1 protein levels were the same in the wild type and *RNA Pol II 140(A5)/+; dcr-2 (G173E)/+*, thus confirming that HP1 is mislocalized and not upregulated in the trans-heterozygote mutants (Additional file 3). The mislocalization can be attributed to a reduced H2K9me2 deposition at the chromocentres of mutants, which allow HP1 to associate with various low affinity binding sites. The above experiments also highlight the role of small RNAs generated by the transcription of heterochromatic repeats in guiding heterochromatin modifications (H3K9me2 and HP1) at the chromocentre.

**Figure 6:**
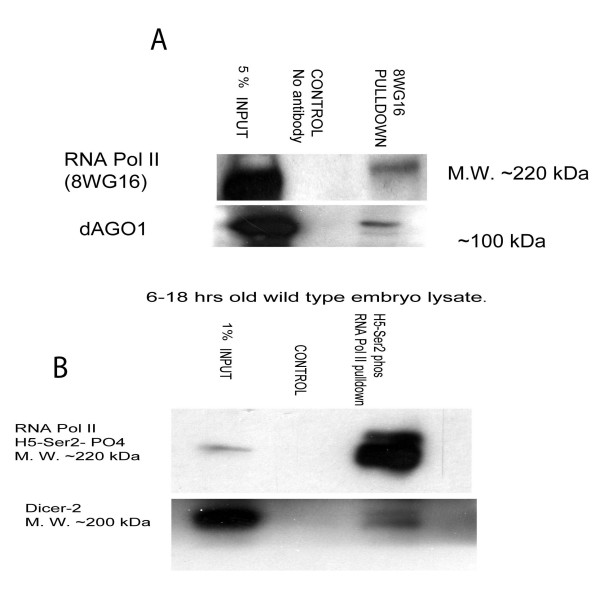
**Biochemical interaction between the RNA Pol II carboxyl terminal domain and RNA silencing machinery components**. (A) Whole cell extracts from 6-18 h wild type embryos were prepared. Native mouse serum was used in the control lane and 8WG16 monoclonal RNA Pol II antibody was used for pull down analysis. About 500 micrograms of lysate was used. AGO1 and Dicer-2 polyclonal antibodies (1: 1000) were used to perform western blot analysis. (B) Co-immunoprecipitation analysis performed similarly as above with H5 antibodies specific for RNA Pol II ser-2 phos.

In order to gain a further insight into the mechanism by which RNA Pol II and RNA silencing machinery regulate heterochromatin structure, we performed co-immunoprecipitation using extracts from *Drosophila *wild type embryos (6-18 h old). The specificity of Dicer-2 antibody was confirmed by western blot analysis (Additional file 4). We found co-IP between Dicer-2 and RNA Pol II ser-2 phos CTD, which is a transcriptionally competent form (Figure 6). This result suggests that the genetic interactions described above have a basis in a biophysical interaction.

In plants, experiments have implied a role of WG/GW motifs as docking sites for Argonaute binding such as for AGO4 to the CTD of the largest subunit of Pol IVb subunit NRPD1B [24], which is a specialized RNA pol II involved with transcriptional silencing. The CTD of the *Drosophila *lacks any reiterative GW/WG motifs and it might be speculated that the absence of these domains could contribute to the lack of any physical interaction between dAGO2 and RNA Pol II. We could not detect any interaction between dAGO2 and RNA Pol II CTD, implying that the interaction is very weak or indirect. The analysis of the amino acid sequence of RNA Pol II 140 revealed the presence of a PxVxV site (residues 350-354) (Additional file 5). Similarly, AGO2 also contains the pentapeptide PxVxV (residues 486-490)(Additional file 5). The peptide sequence PxVxM/L/V represents the conserved sequence found in all HP1 interacting proteins [25]. Recent experiments performed in flies demonstrate that PIWI interacts physically with the HP1 protein by virtue of the presence of the PxVxV domain [26]. The amino acid replacement of the central valine residue of the pentapeptide abolished interaction between HP1 and PIWI, thus highlighting the importance of the pentapeptide domain for this interaction. As RNA Pol II 140 possesses a PxVxV domain, it is interesting to speculate that HP1 might bridge PIWI and AGO2 with the RNA Pol II 140 subunit. This might constitute a novel RNA Pol II complex in metazoans exclusively dedicated for silencing. We could not address this issue because of the unavailability of suitable RNA Pol II 140 antibodies for immunoprecipitation.

However, we found dAGO1, which typically binds miRNAs, co-immunoprecipitated with RNA Pol II (8WG16) CTD antibody (Figure 6), but not with antibodies against the activated CTD. The presence of dAGO1 in the pulldown fraction using 8WG16 antibodies prompted us to investigate the role of the miRNA machinery in heterochromatin modifications. *dcr-1 *and *ago-1 *are two genes that play a predominant role in miRNA metabolism in flies [27,28]. There was no effect on H3K9me2 modification at the chromocentre of polytene chromosomes of *RNA Pol II(A5)/+; dcr-1(Q1147X) *compared to wild type (Additional file 6). Similarly this trans-heterozygote combination did not relieve the silencing of the *mini-white *array DX1 or the *In(1)w [m4h] *heterochromatin environment. When trans-heterozygotes of *RNA Pol II140 (A5)/+; ago1(k04845)/+ *were introduced into the *In(1)w [m4h] *background, there was moderate suppression of PEV (Additional file 7). Similarly, the chromocentre of polytene chromosomes in this background caused moderate reduction of H3K9me2 compared to wild type and there was a significant reduction in H3K9me2 levels in the Western blot analysis. The presence of AGO1 in the RNA Pol II (8WG16) pulldown fraction indicates that AGO1 might have an affinity for binding to small RNAs arising from heterochromatin. This may well be the case as there is evidence that AGO1 and AGO2 have somewhat overlapping functions and there is sharing of biochemical components among miRNA, endo-siRNA and siRNA pathways in *Drosophila *[29,30].

**Figure 7:**
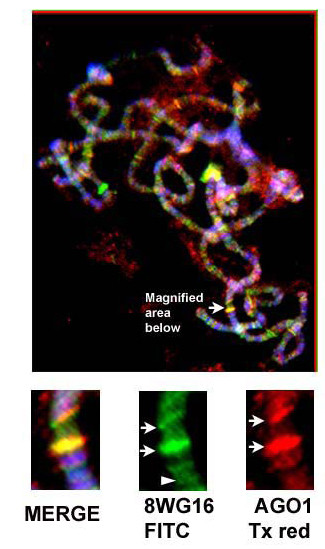
**Co-localization of RNA Pol II and AGO1 on polytene chromosomes**. High magnification of the overlap sites between AGO1 (red) and 8WG16 (green). Arrows indicate sites of co-localization and arrow head indicates RNA Pol II site not overlapping with 8WG16. Third instar larvae from Canton S flies were used.

In order to address the *in vivo *association between RNA Pol II and small RNA silencing machinery proteins, we examined possible co-localization patterns between them on polytene chromosomes. The immunofluorescence analysis on polytene chromosomes revealed a few sites of co-localization between RNA Pol II (8WG16 antibody) and dAGO1 (Figure 7 and Additional files 8 and 9). The overlapping positions between AGO1 and 8WG16 (RNA Pol II) might potentially represent sites where the small RNA machinery is involved with RNA Pol II in maintaining local chromatin structure and hence gene expression. The *in vivo *association between RNA Pol II and AGO1 at few sites on polytene chromosomes provides further evidence of a physical association between RNA Pol II and the small RNA silencing machinery. The association of RNA Pol II with PIWI, AGO-2 and Dicer-2 could not be addressed because of the non-availability of antibodies suitable for polytene chromosome staining.

The TAF-1/TFIID (TATA box binding protein associated factor 1) is the major component of the transcription initiation complex in eukaryotes. Trans-heterozygotes of *RNA Pol II140(A5)/+; TAF-1/+ *and the single heterozygote *TAF-1/+ *shows no effect on suppression of PEV (Additional file 10). This control shows that mutations affecting transcription factors (TAF-1 interacts with RNA Pol II as part of general transcription machinery) had no effect on suppression of PEV and implicates the specific interaction of pol II with the small RNA silencing components.

In order to test whether the double heterozygous combinations affect post-transcriptional functions of the RNA silencing machinery, selected genotypes were examined for an effect on *white *RNAi. The trans-heterozygotes of (1) *RNA Pol II(A5)/+; piwi[1]/+*; (2) *RNA Pol II (A5)/+; hls [125]/+*; and (3) *RNA Pol II (A5)/+; dcr-2(L811fsX)/+ *had no effect on w-IR RNAi (Additional file 11). This experiment indicates that in *RNA Pol II 140(A5)/+ *heterozygotes in which the dose of RNA Pol II is halved, the effect is more pronounced on the heterochromatin structure (transcriptional gene silencing (TGS), but the w-IR RNAi pathway is unaffected under these circumstances. To obtain further insight into the role of RNA Pol II in silencing, we tested for an effect on TGS silencing involving the interaction between *Alcohol dehydrogenase-white *hybrid transgenes [31]. In these flies the transgene copies of *w-Adh *bring about silencing of *Adh-w *at the transcriptional level; however, the silencing is eliminated in a *piwi *mutant background [31,32]. When *RNA Pol II 140(A5)/+ *was introduced into this genotype, there was no apparent effect on silencing (Additional file 12). The trans-heterozygotes of *RNA Pol II 140(A5)/+; hls [125]/+ *also had no effect on relieving the silencing of *Adh-w *by *w-Adh *transgenes.

## Conclusion

The role of small RNAs in maintaining genomic stability and chromosome structure is receiving increasing attention. It has now been well articulated that in *Drosophila, S. pombe *and mouse defects in the RNAi machinery lead to a compromised heterochromatin structure and aberrant regulation of transposable elements. Recent studies in *S. pombe, Arabidopsis *and mouse have further elucidated the mechanism by which small RNAs arising from heterochromatic repeat transcription modify histone methylation in the heterochromatin. Plants have an exclusive RNA Pol V dedicated for heterochromatin maintenance [33,34]. The case of centromeric heterochromatin formation in *S. pombe *is best studied in regard to the relationship between the RNAi machinery and RNA Pol II transcription.

We observed genetic and biochemical interaction between the second largest subunit of RNA Pol II and RNA silencing genes. The genetic interaction had a chromatin component in terms of suppressing PEV and reduction of H3K9me2 levels. The interaction between RNA Pol II and Dicer-2 was the strongest in terms of suppressing PEV of *w [m4h]*. In contrast, *dcr-1*, which has a key role in miRNA metabolism, had no role in determination of heterochromatin structure in our studies. The interaction of RNA Pol II with other proteins in the RNA silencing pathway displayed a similar level of effect on heterochromatin structure.

An interesting aspect of this study is the involvement of *piwi *and *aub *in suppressing PEV and H3K9me2 reduction in combination with RNA Pol II. PIWI and Aub associate with a unique class of rasiRNAs called piRNAs that are found predominantly in the germline cells and are independent of the Dicer processing machinery [15,35]. However, *piwi *mutations also have effects in somatic cells and the PIWI protein has been demonstrated to be present in the soma and piRNAs have been detected in somatic tissues [26,36]. Also, the fact that Dicer-2 co-immunoprecipitates with RNA Pol II and their genetic interaction strongly suppress PEV might indicate a possible role for endo-siRNAs in regulating heterochromatin structure.

Recent experiments in plants reveal the presence of a RNA Pol V complex which shares some subunits of RNA Pol II and participates in RNA silencing [37]. Because animals lack RNA Pol IV and Pol V, it is possible that they possess a specialized RNA Pol II complex involved in RNA mediated silencing. The interaction of Dicer-2 with the transcriptionally competent form of RNA Pol II might reflect one such form. It might also represent an additional mechanism of gene regulation by degrading aberrant transcripts during the elongation process. The involvement of dAGO1 with RNA Pol II in co-immunoprecipitation experiments suggests an additional level of complexity regarding the involvement of 'Argonaute slicer' in the cleavage of small RNAs. While this result might implicate miRNAs being involved in heterochromatin formation, the following observations suggest otherwise: (1) trans-heterozygotes of RNA Pol II and *dcr-1 *have no effect on *w [m4h] *PEV suppression and H3K9me2 levels; and (2) miRNAs have a central bulge making it difficult to explain how specificity is achieved when miRNAs base pairs imperfectly with nascent centromeric transcripts/centromeric DNA. The involvement of AGO1 probably suggests that small RNAs arising out of cleavage of nascent heterochromatic transcripts have some affinity for AGO1.

Our experiments suggest that RNA Pol II transcription through centromeric heterochromatin results in a nascent transcript that could fold into a stem loop structure by virtue of the presence of many direct and inverted repeats present in the heterochromatin. This acts as a substrate for Dicer-2 generating endo-siRNAs, which guide chromatin modifications at the heterochromatin. The interaction of Dicer with RDRC has been shown to be crucial for centromeric heterochromatin structure in *S. pombe*. The interaction between RNA Pol II and Dicer-2 in *Drosophila *reflects a similar mechanism. The exact role of the RNA Pol II second largest subunit mutations on small RNA synthesis from the centromere is not known but it is possible that it disrupts the biochemical interaction between the CTD of the largest subunit and Dicer-2. The heterochromatin specific histone modifications are dependent on both the RNA pol II complex as well as small RNA silencing machinery components.

## Methods

### Fly stocks and PEV analysis

All flies were grown at 25°C on standard food medium. The genotypes were:

y w; RNA Pol II 140(A5)/TM3, Sb

RNA PolI 215(W81)/FM7a

ru[1] h[1] th[1] st[1]cu[1]RpII140 [wimp] sr[1[ e [s] ca[1]/TM3, Sb[1]

y w; ago2(414)

y w; eyFLP; FRT 42 D dcr-2(G173E)/CyO

y w; eyFLP FRT 42 D dcr-2(L811fsX)/CyO

y w; eyFLP FRT 82B dcr-1(Q1147X)/TM3, Ser

cn1 P(ry t 7.2) ago-1(04845)/CyO

ru st hls [E616] e ca/TM3, Sb e

ry cv c sbd hls [DE8]/TM2, Ubx ry e

*w*^1^; *hls [125] e/TM3, Sb e*

aub [QC42]/CyO

*w*^1^; *aub Df(P3a)/CyO*

y w; piwi[1]/CyO

y w; piwi[2]/CyO

y w; DX1/CyO

The male flies of each of the genotypes mentioned above were crossed to female *In(1)w [m4h]; SM6a/Gla; TM3, Sb/Ser*. The single heterozygotes of *RNA Pol II 140 *in the *w [m4h] *background were then mated with RNA silencing mutants in a similar background. The F2 males were then sorted into different single and double heterozygous groups and analysed for PEV.

In order to analyse the effect of *piwi[1] *on DX1 silencing, the DX1(lac w) transgene array was recombined with Sco and balanced over CyO to produce *DX1 Sco/CyO *flies. The males of *DX1 Sco/CyO *were then crossed to female *piwi[1]/CyO*. In the next generation, female non-curly *DX1 Sco/piwi[1] *females were selected, which then were crossed to male *w; Gla/SM6a; TM3, Ser/Sb*. In the next generation non-Sco flies *DX1 piwi[1]/CyO *were obtained.

### Measurement of eye pigment

Fly heads from 10 animals were homogenized in methanol containing 0.1% hydrochloric acid (HCl). The absorbance of the supernatant was measured at 480 nm after the centrifugation of the fly head homogenate. Three independent experiments were performed in each case.

### Immunofluoresence analysis of polytene chromosomes

To analyse trans-heterozygotes of *RNA Pol II 140 and dcr-2, piwi *and *hls*, we crossed them into a *T(2;3)CyO Tb *background. In the next step, males of *RNA Pol II 140 *and females of *piwi[1], dcr-2(G173E), hls [125*] (each balanced over *T(2;3) CyO Tb*) were crossed and non-Tb third instar larvae were selected for further analysis. Three to four pairs of salivary glands each from control and trans-heterozygotes were dissected in 0.7% NaCl. The glands were then fixed for about a minute in 3.7% formaldehyde in phosphate buffered saline (PBS; ice-cold). The glands were then kept in a solution of 45% acetic acid and 3.7% formaldehyde for about 2 min and then squashed. The slide was placed on dry-ice for 20 min, the cover slip removed and then washed twice in PBS for 10 min each and blocked for 30 min in a solution of PBS containing bovine serum albumin (BSA). The following antibodies were used at 1:100 dilutions: Sxl (Hybridoma bank, University of Iowa, USA), HP1 (from Dr S Elgin) and H3K9me2 (Upstate). The primary antibodies were incubated overnight at 4°C. On the following day, the slides were washed twice in PBS and blocked in PBS-BSA solution. The slides were then blocked with 5% goat serum for 30 min at 37°C. The secondary antibodies (1:100 goat anti rabbit-conjugated with fluorescein isothiocyanate and 1:200 goat anti-mouse conjugated with Texas red) were then applied to the slide for about 1 h at 37°C. The slides were washed twice in PBS and visualized using fluorescence microscopy after application of 4', 6-diamidino-2-phenylindole. The images were adjusted using Photoshop CS3 version software.

The gently squashed polytene spreads [38] were prepared in the same manner except that solution II (45% acetic acid in PBS) was omitted. The glands were squashed in 0.7% NaCl and the fixed in 3.7% formaldehyde for 20 min at 4°C.

### Western blot analysis

Adult flies (aged 12-15) were dissected to remove the ovaries. The carcasses were then homogenized in HEPES buffer containing protease inhibitor cocktail (Pierce). The homogenate was then acidified with HCl to a final concentration of 0.2 N HCl and kept on ice for 1 h. The homogenate was then centrifuged at 11,000 g for 15 min and the supernatant was then neutralized with NaOH. The histone enriched protein lysate was then boiled with Laemmli sodium dodecyl sulphate sample buffer and loaded onto the gel. The Western blot analysis was performed by standard procedures [23]. The antibodies used were rabbit polyclonal H3K9me2 (1:1000) and H4 loading control (1:1000). Supersignal pico chemiluminescent substrate kit (Pierce) was used to observe the bands and Image gauge (NIH) software was used to measure the density of bands.

### Co-immunoprecipitation

Wild type embryos at 6-18 h were homogenized in total lysis buffer containing 1 M Tris pH 8, 150 mM NaCl, 10 mM EDTA, 10% glycerol and protease inhibitor cocktail (Pierce). The homogenate was then kept for 15 min on ice. After centrifugation the lysate (about 500 μg) was applied to the activated amino link resin beads. The beads had RNA Pol II monoclonal antibodies 8WG16 (50 μg from Covance) covalently linked to the beads. The lysate was incubated with the beads with gentle mixing at 4°C overnight. The interacting proteins were eluted and analysed by western blots. Native mouse serum was used as a negative control. The antibodies used were Dicer-2 and Ago-1 rabbit polyclonal antibodies.

### Immuno-co-localization using blocking peptide on polytene chromosomes

Salivary glands from wild type larvae were squashed and fixed in formaldehyde and acetic acid solutions as described above. AGO1 antibody (Abcam) was diluted 1:4 in PBT (PBS+Triton X-100). In order to test for the specificity of the antibody the specific peptide bound by the AGO1 antibodies was used at 1:50 dilution, mixed with AGO1 and incubated at room temp for 30 min with occasional gentle shaking. Sxl antibody (Hybridoma bank, Iowa, USA) was used as an internal control. The nuclei were observed by standard methods as described above.

### Chromatin Immunoprecipitation

The ChIP method was adapted from a previously published protocol [39]. For each ChIP, a reference sample (Mock) corresponded to a ChIP performed at the same time without the addition of the specific antibody. Formaldehyde, at a final concentration of 1.8% in buffer A1, that is, 60 mM KCl, 15 mM NaCl, 4 mM MgCl2, 15 mM HEPES (pH 7.6), 0.5% Triton X-100, 0.5 mM DTT, 10 mM sodium butyrate, protease inhibitor cocktail (Roche, Basel, Switzerland), was used for crosslinking while crushing whole *Drosophila *animals (adults) for 10 min at room temperature. After blocking the reaction with glycine, and after three washes (5 min each at 4°C with buffer A1), subsequent steps were performed as described in [39]. For immunoprecipitation (IP) reactions, 5 μl H3K9me2 (Millipore) was used per reaction.

### Real time polymerase chain reaction analysis

Two microlitresl of DNA sample was used for the analysis. All samples were analysed in triplicate. The threshold values (Ct) were used to calculate the fold change differences by the delta delta Ct method. The IP and mock samples were normalized to the input. The ABI 7300 (Applied Biosystems Inc) was used for the analysis. The SYBR green master mix was purchased from Applied Biosystems Inc. The primer sequences used were:

tubulin-

forward-5'AGCAAATTACTTGCAGAATTGG3'

reverse-5'GATTAGTGCGATTAGGACTTG3'

white-

forward-5'CAATCACCACCCCAATCACTC3'

reverse-5'TCCGCTATCTCTTTCGCCAC3'

## Abbreviations

BSA: bovine serum albumin; ChIP: chromatin immunoprecipitation; CTD: carboxyl terminal domain; HCl: hydrochloric acid; HP1: heterochromatin protein-1; IP: immunoprecipitation; PBS: phosphate buffered saline; PEV: position-effect variegation; RDRC: RNA-dependent RNA polymerase complex; RITS: RNAi-induced transcriptional silencing; TGS: transcriptional gene silencing.

## Competing interests

The authors declare that they have no competing interests.

## Authors' contributions

HK and JB designed the experiments; HK performed the experiments; HK and JB wrote the paper.

## Supplementary Material

Additional file 1 **Position-effect variegation (PEV) analysis of male flies of small RNA and RNA Pol II mutations**. The trans heterozygote of RNA Pol II and small RNA pathway mutations showed very strong suppression of *In(1)w [m4h] *PEV compared with control and single heterozygote mutants. All the male flies were of same age (4 days after eclosion). Click here for file

Additional file 2 **Immunofluorescence analysis of polytene chromosomes in RNA Pol II and small RNA pathway trans-heterozygote mutants**. H3K9me2 modification is strongly reduced in trans-heterozygotes compared with the control. The FITC (green) channel shows H3K9me2 antibody signal while the Texas red shows Sxl antibody signal. Representative images from five different experiments (approx 50 pairs of nuclei) have been examined. The genotypes of each polytene nucleus has been indicated. Click here for file

Additional file 3 **Western blot analysis of heterochromatin protein-1 (HP1) in small RNA and RNA Pol II trans-heterozygote mutants**. Western blot analysis with HP1 and tubulin (loading control) antibodies on adult carcasses of the indicated genotypes. No significant upregulation of HP-1 was observed in mutants compared with wild type. The standard error bars were calculated from three different experiments. Click here for file

Additional file 4 **Western blot analysis to check the specificity of the dcr-2 antibody**. The western blot analysis performed on third instar larvae shows the absence of the specific band at ~200 kDa in *dcr-2 (L811fsX)*. Click here for file

Additional file 5 **Amino acid sequence of RNA Pol II second largest subunit and dAgo-2**. The consensus heterochromatin protein-1 binding pentapeptide sequence (PxVxV) is highlighted in bold letters. Click here for file

Additional file 6 **Eye pigment analysis of *dcr-1(Q1147X) *and *RNA Pol II 140(A5) *in *w [m4h] *background**. Trans-heterozygotes of *dcr-1(Q1147X) *and *RNA Pol II 140(A5) didn't* affect position-effect variegation. Three independent replicas were performed. Standard error is shown. The genotypes of male flies are indicated. Click here for file

Additional file 7 **Role of miRNA machinery in heterochromatin formation**. Immunofluorescence analysis of polytene chromosomes using H3K9me2 (FITC) and Sxl (Tx red) antibodies on the noted genotypes. Click here for file

Additional file 8 **Co-localization of AGO1 and RNA Pol II (8WG16) on polytene chromosomes**. The arrows indicate sites of co-localization between AGO1 and RNA Pol II. Canton S wild type third instar larvae were used. Click here for file

Additional file 9 **Analysis of AGO1 localization**. The upper panels show AGO1 and Sex lethal proteins on female polytene chromosomes. The lower panel is the control experiment demonstrating the specificity of the antibody using AGO1 specific blocking peptide. Sxl is the internal control and it is unaffected by AGO1 blocking peptide. Click here for file

Additional file 10 **Effect of *Taf-1 *(TATA Box Associated factor 1) on position-effect variegation using *In(1)w [m4h] *male flies**. All male flies were of the same age (4 days after eclosion). Click here for file

Additional file 11 **Effect of RNA Pol II and RNA silencing machinery on white-IR post transcriptional gene silencing (PTGS)**. Trans-heterozygotes of RNA Pol II 140 and small RNA mutants don't affect *w-IR *PTGS. The genotypes of male flies are indicated. Click here for file

Additional file 12 **Effect of RNA Pol II 140(A5) and small RNA silencing machinery (hls [125]) on *Adh-white *(1 copy) and *white-Adh *(2 copies) female flies**. All flies were observed 2 h after eclosion. Click here for file
