# Supplementary material for: Interaction of RNA polymerase II and the small RNA machinery affects heterochromatic silencing in Drosophila
Source: Epigenetics Chromatin. 2009 Nov 16;2:15. doi: 10.1186/1756-8935-2-15 (PMC2785806; doi:10.1186/1756-8935-2-15)
Supplement: Additional file 1 — Position-effect variegation (PEV) analysis of male flies of small RNA and RNA Pol II mutations. The trans heterozygote of RNA Pol II and small RNA pathway mutations showed very strong suppression of In(1)w [m4h] PEV compared with control and single heterozygote mutants. All the male flies were of same age (4 days after eclosion). [file 1756-8935-2-15-S1.PDF]

SM6a/+;RNA Pol II140(A5)/+

aub[QC42]/+;
RNA Pol II140(A5)/+

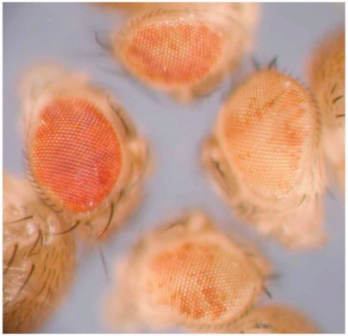

SM6a/+;MKRS/TM3,Ser
control

aub[QC42];MKRS/TM3,Ser

MKRS/TM3,Ser
control

hls[E616]/+;
RNA Pol II140(A5)/+

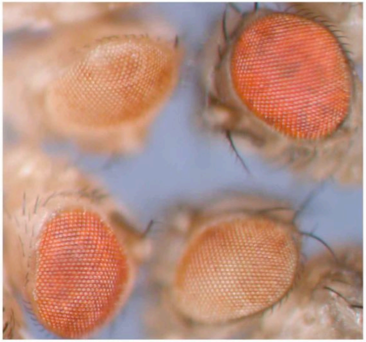

RNA Pol II140(A5)
/MKRS

hls[E616]/
TM3,Ser

RNA Pol II140(A5)/TM3,Ser

hls (DE8)/ MKRS

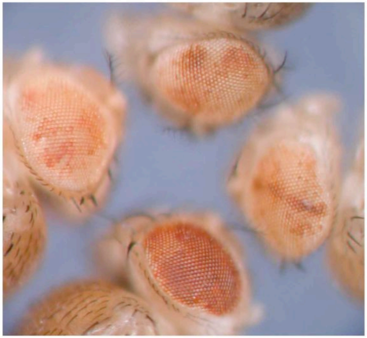

MKRS/TM3,Ser
control

hls (DE8)/+;RNA Pol II140(A5)/+

hls[125]/+;
RNA Pol II140
(wimp)/+

MKRS/TM3,Ser
control

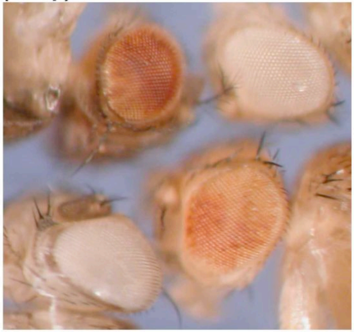

hls[125]/
TM3,Ser

RNA Pol II140
(wimp)/+

ago-2[414]/MKRS

MKRS/TM3,Ser
CONTROL

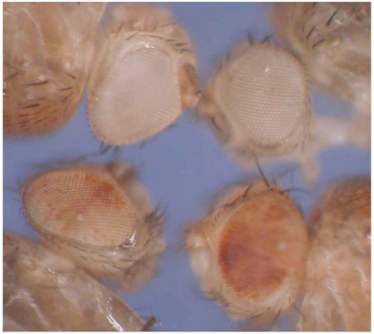

SM6a/+;MKRS)/TM3,Ser

RNA Pol II140
(wimp)/Ser

ago-2[414]/+;
RNA Pol II140(wimp)/+

dcr-2(L811fsX)/+;
MKRS/TM3,Ser

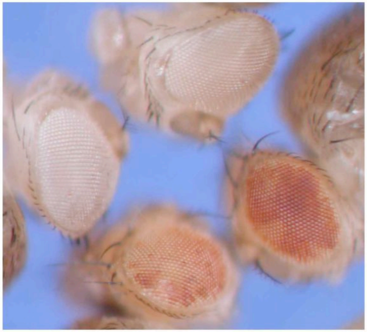

dcr-2(L811fsX)/+;
RNA Pol II140(A5)/+

SM6a/+;RNA Pol II140(A5)/+
