# Supplementary material for: Interaction of RNA polymerase II and the small RNA machinery affects heterochromatic silencing in Drosophila
Source: Epigenetics Chromatin. 2009 Nov 16;2:15. doi: 10.1186/1756-8935-2-15 (PMC2785806; doi:10.1186/1756-8935-2-15)
Supplement: Additional file 2 — Immunofluorescence analysis of polytene chromosomes in RNA Pol II and small RNA pathway trans-heterozygote mutants. H3K9me2 modification is strongly reduced in trans-heterozygotes compared with the control. The FITC (green) channel shows H3K9me2 antibody signal while the Texas red shows Sxl antibody signal. Representative images from five different experiments (approx 50 pairs of nuclei) have been examined. The genotypes of each polytene nucleus has been indicated. [file 1756-8935-2-15-S2.PDF]

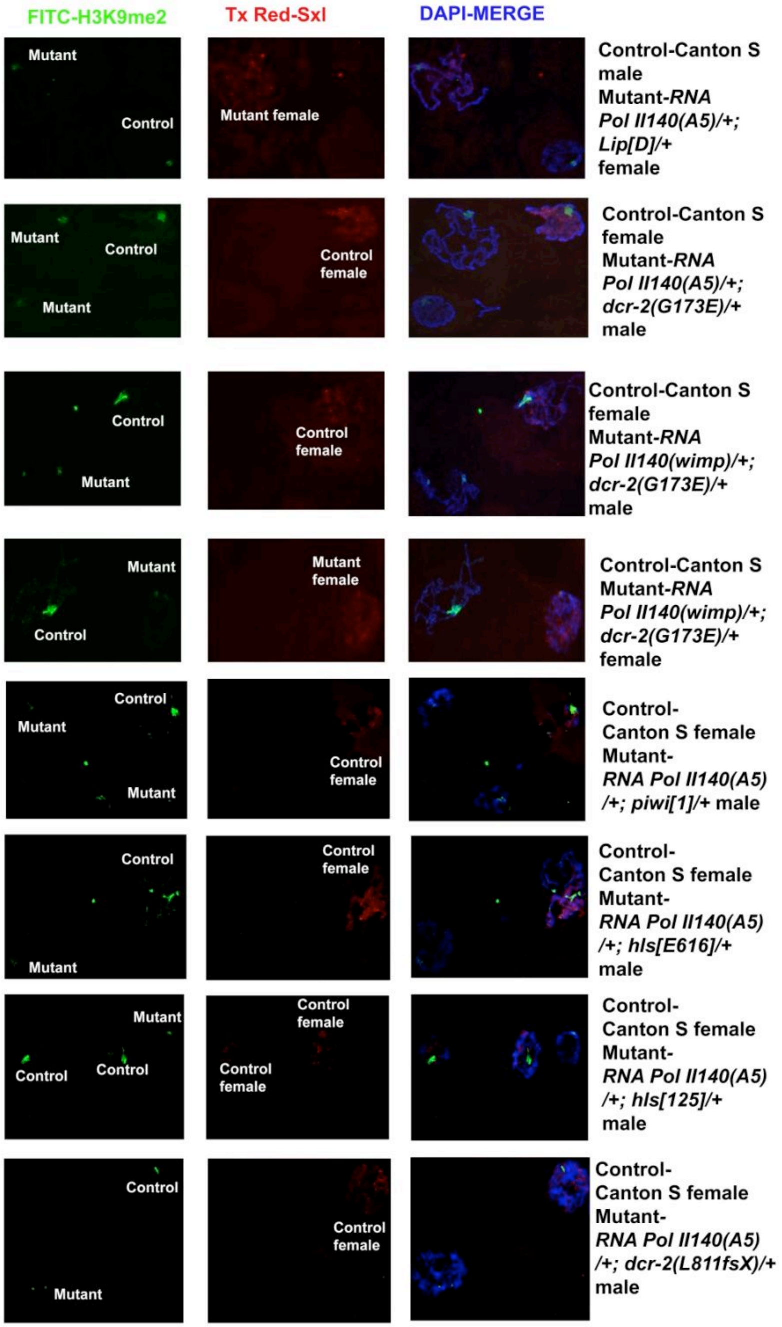
FITC-H3K9me2
Tx Red-Sxl
DAPI-MERGE
Mutant
Control
Mutant female
Control-Canton S male
Mutant-*RNA Pol II140(A5)/+; Lip[D]/+* female
Mutant
Control
Mutant
Control female
Control-Canton S female
Mutant-*RNA Pol II140(A5)/+; dcr-2(G173E)/+* male
Control
Mutant
Control female
Control-Canton S female
Mutant-*RNA Pol II140(wimp)/+; dcr-2(G173E)/+* male
Mutant
Control
Mutant female
Control-Canton S
Mutant-*RNA Pol II140(wimp)/+; dcr-2(G173E)/+* female
Control
Mutant
Mutant
Control female
Control-Canton S female
Mutant-*RNA Pol II140(A5) /+; piwi[1]/+* male
Control
Mutant
Control female
Control-Canton S female
Mutant-*RNA Pol II140(A5) /+; hls[E616]/+* male
Mutant
Control
Control
Control female
Control female
Control-Canton S female
Mutant-*RNA Pol II140(A5) /+; hls[125]/+* male
Control
Mutant
Control female
Control-Canton S female
Mutant-*RNA Pol II140(A5) /+; dcr-2(L811fsX)/+* male
