# Supplementary material for: Interaction of RNA polymerase II and the small RNA machinery affects heterochromatic silencing in Drosophila
Source: Epigenetics Chromatin. 2009 Nov 16;2:15. doi: 10.1186/1756-8935-2-15 (PMC2785806; doi:10.1186/1756-8935-2-15)

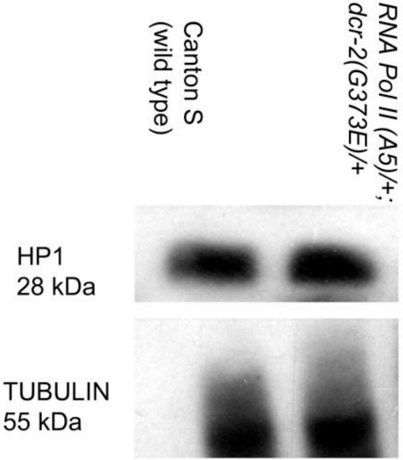

Western blots on adult flies

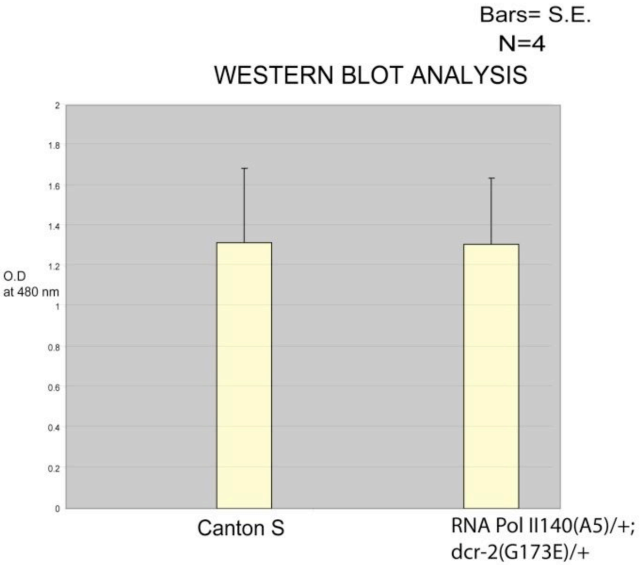

Supplement: Additional file 3 — Western blot analysis of heterochromatin protein-1 (HP1) in small RNA and RNA Pol II trans-heterozygote mutants. Western blot analysis with HP1 and tubulin (loading control) antibodies on adult carcasses of the indicated genotypes. No significant upregulation of HP-1 was observed in mutants compared with wild type. The standard error bars were calculated from three different experiments. [file 1756-8935-2-15-S3.PDF]
