# Supplementary figures and images for: Interaction of RNA polymerase II and the small RNA machinery affects heterochromatic silencing in Drosophila
Source: Epigenetics Chromatin. 2009 Nov 16;2:15. doi: 10.1186/1756-8935-2-15 (PMC2785806; doi:10.1186/1756-8935-2-15)

250 kDa

~200kDa  
Dicer-2

150kDa

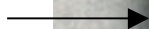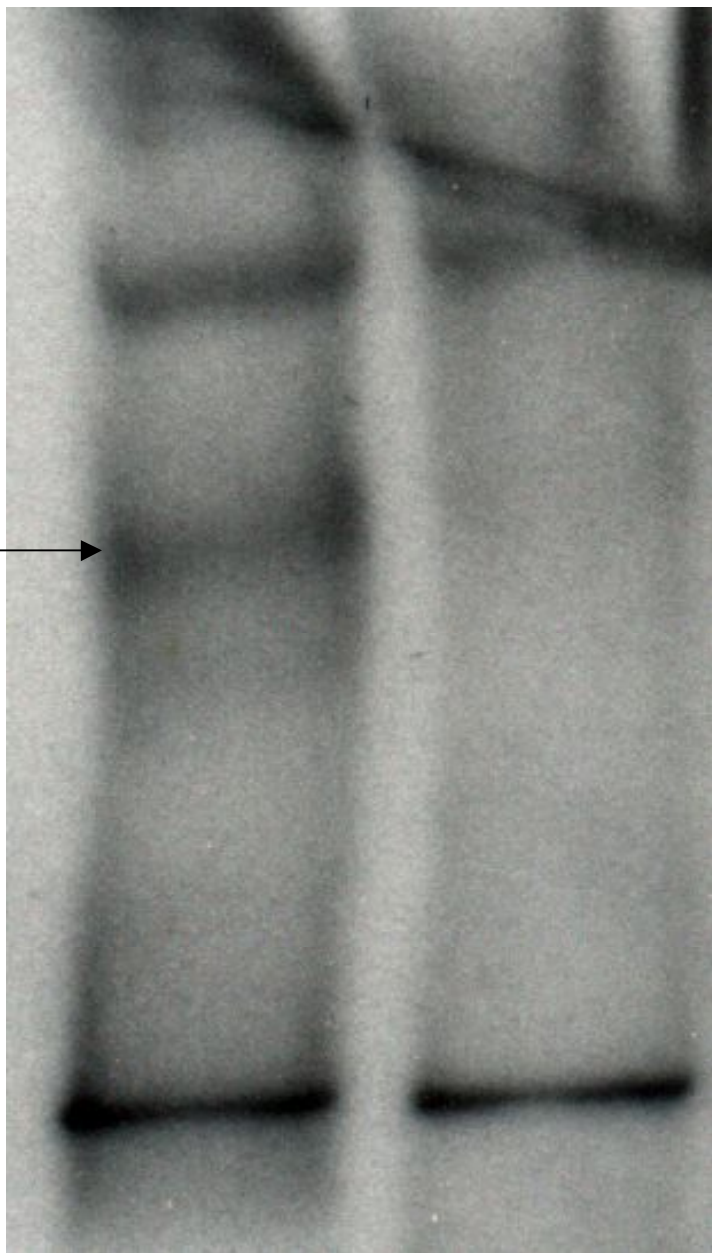

Canton S

*dcr-2(L811fsX)*

Supplement: Additional file 4 — Western blot analysis to check the specificity of the dcr-2 antibody. The western blot analysis performed on third instar larvae shows the absence of the specific band at ~200 kDa in dcr-2 (L811fsX). [file 1756-8935-2-15-S4.PDF]

**MERGE**

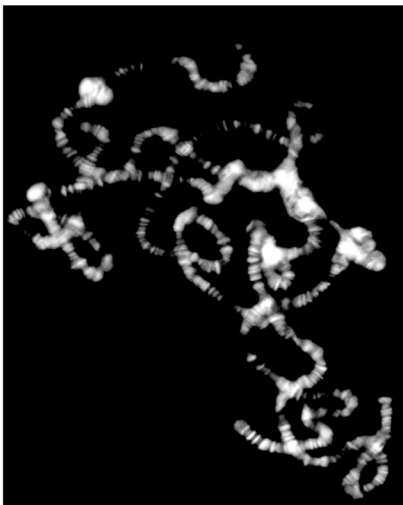

**AGO1**

**8WG16 (RNA Pol II)**

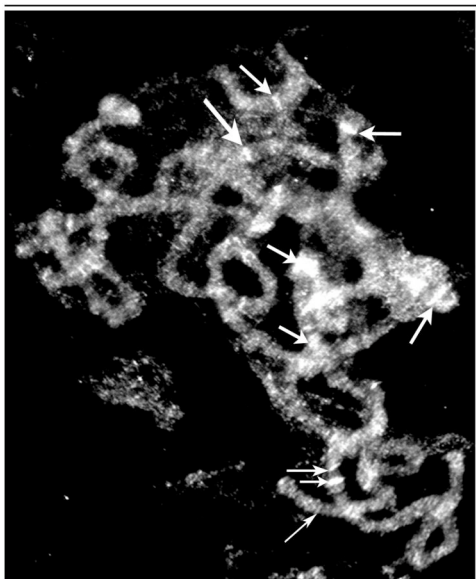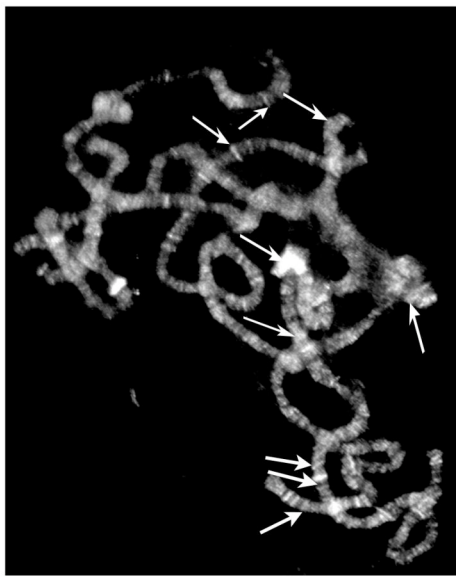

Supplement: Additional file 8 — Co-localization of AGO1 and RNA Pol II (8WG16) on polytene chromosomes. The arrows indicate sites of co-localization between AGO1 and RNA Pol II. Canton S wild type third instar larvae were used. [file 1756-8935-2-15-S8.PDF]

AGO1

Sxl

MERGE

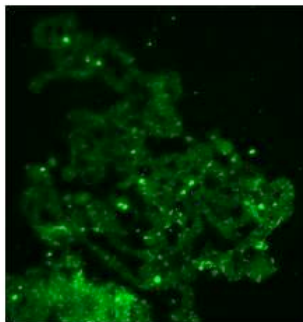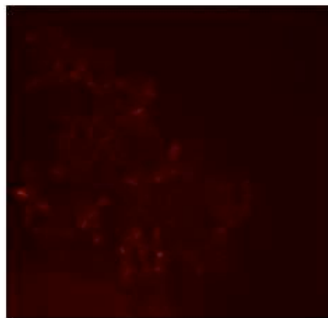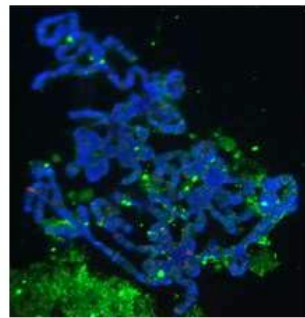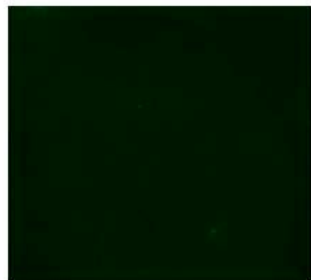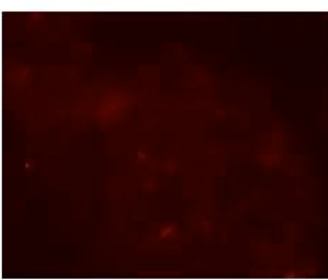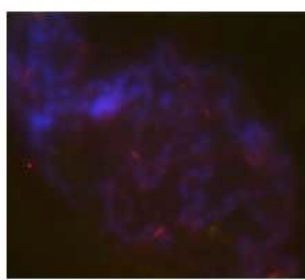

AGO1 +  
blocking  
peptide

Sxl

Tx red

MERGE

Supplement: Additional file 9 — Analysis of AGO1 localization. The upper panels show AGO1 and Sex lethal proteins on female polytene chromosomes. The lower panel is the control experiment demonstrating the specificity of the antibody using AGO1 specific blocking peptide. Sxl is the internal control and it is unaffected by AGO1 blocking peptide. [file 1756-8935-2-15-S9.PDF]

***RNA Pol II140(A5)/+;***  
***Taf [1]/+***

***Taf[1]/MKRS***

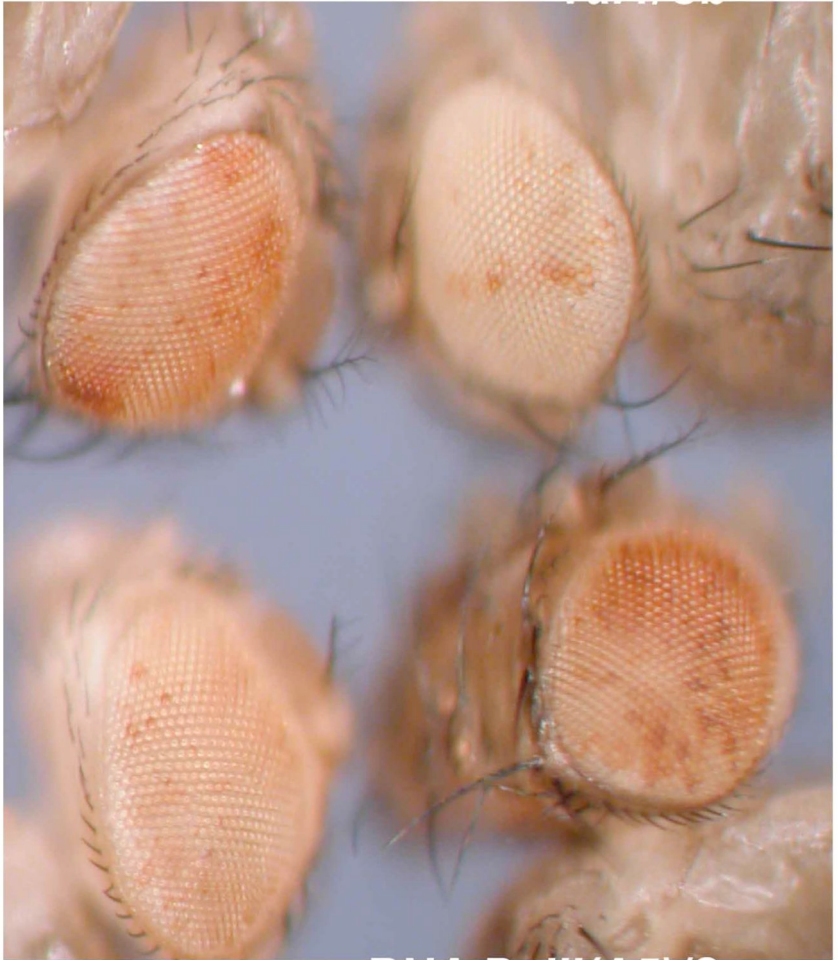

***In(1)w[m4h]***  
***MKRS/TM3,Ser***  
***(control)***

***RNA Pol II140(A5)/+***

Supplement: Additional file 10 — Effect of Taf-1 (TATA Box Associated factor 1) on position-effect variegation using In(1)w [m4h] male flies. All male flies were of the same age (4 days after eclosion). [file 1756-8935-2-15-S10.PDF]
