# Supplementary material for: Interaction of RNA polymerase II and the small RNA machinery affects heterochromatic silencing in Drosophila
Source: Epigenetics Chromatin. 2009 Nov 16;2:15. doi: 10.1186/1756-8935-2-15 (PMC2785806; doi:10.1186/1756-8935-2-15)
Supplement: Additional file 5 — Amino acid sequence of RNA Pol II second largest subunit and dAgo-2. The consensus heterochromatin protein-1 binding pentapeptide sequence (PxVxV) is highlighted in bold letters. [file 1756-8935-2-15-S5.PDF]

A) Amino acid sequence of *Drosophila* RNA Pol II 140. The presence of consensus HP1 binding site (PxVxV) is shown in bold colored letters.

mmydneeelyeeenaeeishelwqeacwivinayfdekglvrqqldsfdefiqmsvqri[illegible]

edspaielqaeaqhtsgevetpprfslkfeqiylskpthwekdgspspmmpnearlrnlt

ysaplyvditktknveglldpvetqhqktfigkipimlrstycllsqltdrdltelnecpl

dpggyfiingsekvliaqekmatntvyvfsmkdgkyafkteirsclehssrptstlwvnm

margsqnikksaigqriiailpyikqeipimivfralgfvadrdilehiiydfddpemme

mvkpsldeafvvqeqnvalnfigargarpgvtkdrikyakeilqkeml**PHVGV**sdfcet

kkayflgymvhrlllaslgrrelddrdhygnkrldlagpllaflfrglfknlmkevrmyt

qkfidrgkdfnlelaiktniitdglryslatgnwgdqkkahqaragvsqvlnrltfastl

shlrrvnspigrdgklakprqlhntlwgmlcpaetpegaavglvknlalmayisvgsqps

hqqqqssrqqpstssggsrasgfqqgqqqksqdaegwtaqkkqgkqqvqgwtkqgqqg

ghqqgrqgqdggyqqrppgqqqgghqqgrqgqeggyqqrppgqqqgghqqgrqgqeggyq

qrpsgqqqgghqqgrqgqeggyqqrppgqqqgghqqgrqgqeggyqqrpsgqqqgghqqg

rqgqeggyqqrppgqqqgghqqgrqgqeggyqqrppgqqqggheqgrqgqeggyqqrpsg

qqqgghqqgrqgqeggyqqrpsgqqqgghqqgrqgqeggyqqrpsgqqqgghqqgrqgqe

ggyqqrppgqqpnqtqsqgqyqsrgppqqqqaaplplppqpagsikrgtigkpgqvginy

ldldlskmpsvayhydvkimperpkkfyrqafeqfrvdqlggavlaydgkascysvdklp

lnsqn**PEVTV**tdrngrtlrytieiketgdstidlkslttymndrifdkpmramqcvevvl

aspchnkairvgrsffkmsdpnnrhelddgyealvglyqafmlgdrpflnvdishksfpi

smpmieylerfslkakinnttnldysrrflepflrginvvytppqsfqsaprvyrvngls

rapassetfehdgkkvtiasyfhsrnyplkfpqlhclnvgssiksillpielcsieegqa

lnrkdgatqvanmikyaatstnvrkrkimnllqyfqhnldptisrfgiriandfivvstr

vlsppqveyhskrftmvkngswrmdgmkflepkpkahkcavlycdprsgrkmnytqlndf

gnliisqgkavnisldsdvtyrpftddersldtifadlkrsqhdlaiviipqfrisydti

kqkaelqhgiltqcikqftverkcnnqtignillkinsklnginhkikddprlpmmkntm

pilefleewsmenleeiapsaiadatkifvngcwvgihrdpeqlmatlrklrrqmdiivs

evsmirdirdreiriytdagricrpllivengslllkkthvemlkerdynnyswqvlvas

gvveyidtleeetvmiamspydlkqdkdyaycttythceihpamilgvcasiipfpdhnq

sprntyqsamgkqamgvyitnfhvrmdtlahvlyypmkplvttrsmeylrfrelpagins

ivailcytgynqedsvilnasavergffrsvfyrsykdsenkrvgdqeenfekphrgtcq

gmrnahydklddgiiapgirvsgddvvigktitlpenddeldsntkrfskrdastflrn

setgivdqvmltlnsegykfckirvrsvripqigdkfasrhgqkgtcgiqyrqedmaftc

eglapdiiinphaipsrmtighliecl qgklgsnkgeigdatpfndavnvqkistflqey

gyhlrgnevmynghtgrkinaqvflgptyyqrlkhmvddkihsrargpvqilvrqpmegr

ardgglrfgemerdcqishgaaqflrerlfevsdpyrvhicnfcgliaianlrnntfeck

gcknktqisqvrlpyaakllfqelmsmniaprlmvt

B) Amino acid sequence of *Drosophila* AGO2. The presence of consensus HP1 binding site (PxVxV) is shown in bold colored letters

mgkkdknkkggqdsaaapqpqqqkqqqrqqqpqqlqqpqqlqqpqqlqqpqqqqqqp

yigadvthpspdqreipsvvgvaashdpygasynmqyrlqrgaleeiedmfsitlehlrv

ykeyrnaypdhiiyyrdgvsdgqfpkikneelrcikqacdkvgckpkiccvivvkrhhtr

ffpsgdvttsnkfnnvdpgtvvdrtivhpnemqffmvshqaiqgtakptrynvientgnl

didllqqltynlchmfprcnrsvsypapaylahlvaargrvyltgtnrfldlkkeyakrt

ivpefmkknpmyfv
