# Supplementary material for: Interaction of RNA polymerase II and the small RNA machinery affects heterochromatic silencing in Drosophila
Source: Epigenetics Chromatin. 2009 Nov 16;2:15. doi: 10.1186/1756-8935-2-15 (PMC2785806; doi:10.1186/1756-8935-2-15)
Supplement: Additional file 6 — Eye pigment analysis of dcr-1(Q1147X) and RNA Pol II 140(A5) in w [m4h] background. Trans-heterozygotes of dcr-1(Q1147X) and RNA Pol II 140(A5) didn't affect position-effect variegation. Three independent replicas were performed. Standard error is shown. The genotypes of male flies are indicated. [file 1756-8935-2-15-S6.PDF]

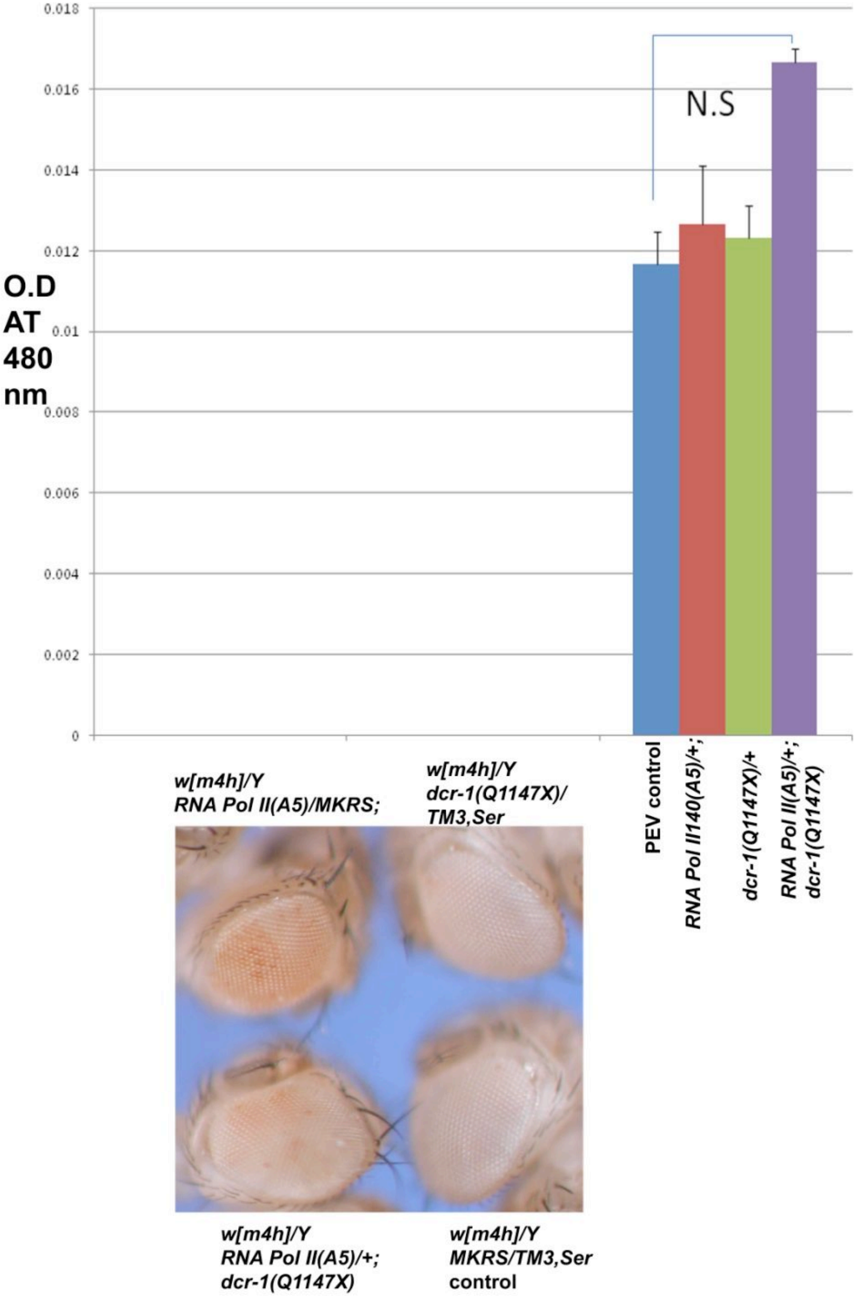

0.018
0.016
0.014
0.012
0.01
0.008
0.006
0.004
0.002
0
O.D AT 480 nm
N.S
PEV control
RNA Pol II140(A5)/+;
dcr-1(Q1147X)/+
RNA Pol II(A5)/+; dcr-1(Q1147X)
w[m4h]/Y RNA Pol II(A5)/MKRS;
w[m4h]/Y dcr-1(Q1147X)/ TM3,Ser
w[m4h]/Y RNA Pol II(A5)/+; dcr-1(Q1147X)
w[m4h]/Y MKRS/TM3,Ser control
