# Supplementary material for: Interaction of RNA polymerase II and the small RNA machinery affects heterochromatic silencing in Drosophila
Source: Epigenetics Chromatin. 2009 Nov 16;2:15. doi: 10.1186/1756-8935-2-15 (PMC2785806; doi:10.1186/1756-8935-2-15)
Supplement: Additional file 7 — Role of miRNA machinery in heterochromatin formation. Immunofluorescence analysis of polytene chromosomes using H3K9me2 (FITC) and Sxl (Tx red) antibodies on the noted genotypes. [file 1756-8935-2-15-S7.PDF]

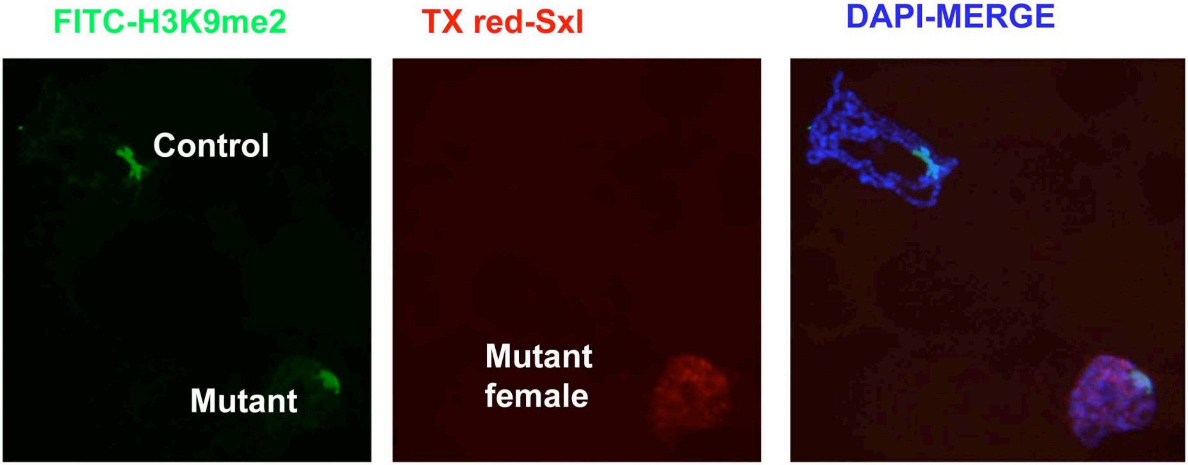

Control-Canton S
Mutant- *RNA Pol II140(A5)/+; ago-1(k04845)/+*

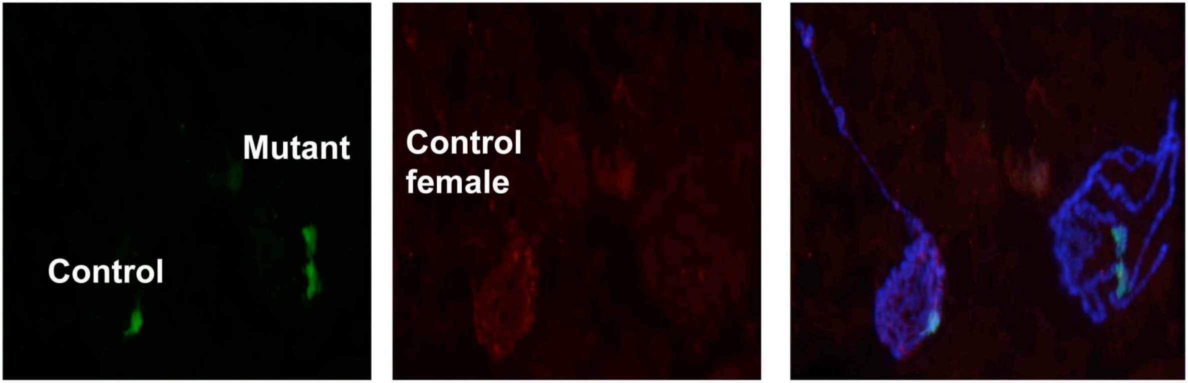

Control-Canton S
Mutant- *RNA Pol II140(A5)/+; dcr-1(Q1141X)*
