# Supplementary material for: Interaction of RNA polymerase II and the small RNA machinery affects heterochromatic silencing in Drosophila
Source: Epigenetics Chromatin. 2009 Nov 16;2:15. doi: 10.1186/1756-8935-2-15 (PMC2785806; doi:10.1186/1756-8935-2-15)
Supplement: Additional file 11 — Effect of RNA Pol II and RNA silencing machinery on white-IR post transcriptional gene silencing (PTGS). Trans-heterozygotes of RNA Pol II 140 and small RNA mutants don't affect w-IR PTGS. The genotypes of male flies are indicated. [file 1756-8935-2-15-S11.PDF]

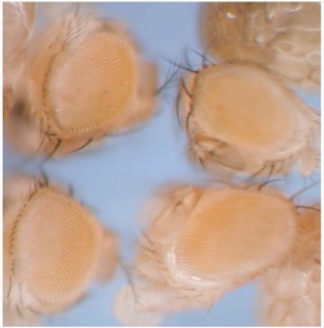
w-IR/Y;
(control)
w-IR/Y; piwi[1]/+;
TM3,Ser/+
w-IR/Y;
SM6a/+;
RNA Pol II140
(A5)/MKRS
w-IR/Y; piwi[1]/+;
RNA Pol II140(A5)/+

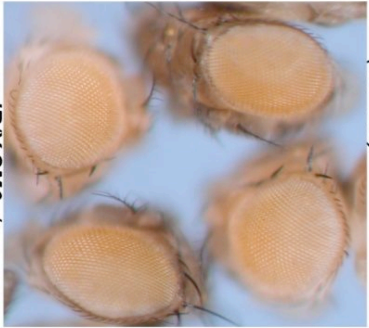
w-IR/Y;SM6a/+
RNA Pol II140(A5)/ +;
hls[125]/+
w-IR/Y;SM6a/+
hls[125]/ MKRS
w-IR/Y;MKRS/Ser
(control)
w-IR/Y;SM6a/+
RNA Pol II140(A5)/ TM3,Ser

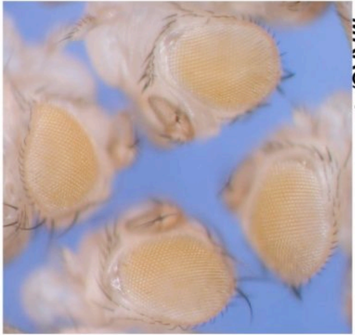
w-IR/Y;SM6a/+
MKRS/ +(control)
w-IR/Y;SM6a/+
RNA Pol II140(A5)/ +
w-IR/Y;dcr-2(L811fsx)/+
MKRS/ +
w-IR/Y;dcr-2(L811fsx)/+
RNA Pol II140(A5)/ +
