# Supplementary material for: Interaction of RNA polymerase II and the small RNA machinery affects heterochromatic silencing in Drosophila
Source: Epigenetics Chromatin. 2009 Nov 16;2:15. doi: 10.1186/1756-8935-2-15 (PMC2785806; doi:10.1186/1756-8935-2-15)
Supplement: Additional file 12 — Effect of RNA Pol II 140(A5) and small RNA silencing machinery (hls [125]) on Adh-white (1 copy) and white-Adh (2 copies) female flies. All flies were observed 2 h after eclosion. [file 1756-8935-2-15-S12.PDF]

Adh-w/w; w-Adh/w-Adh;hls[125]/+, RNA Pol II(A5) 140/+

Adh-w/w; w-Adh/ w-Adh; hls[125]/ MKRS

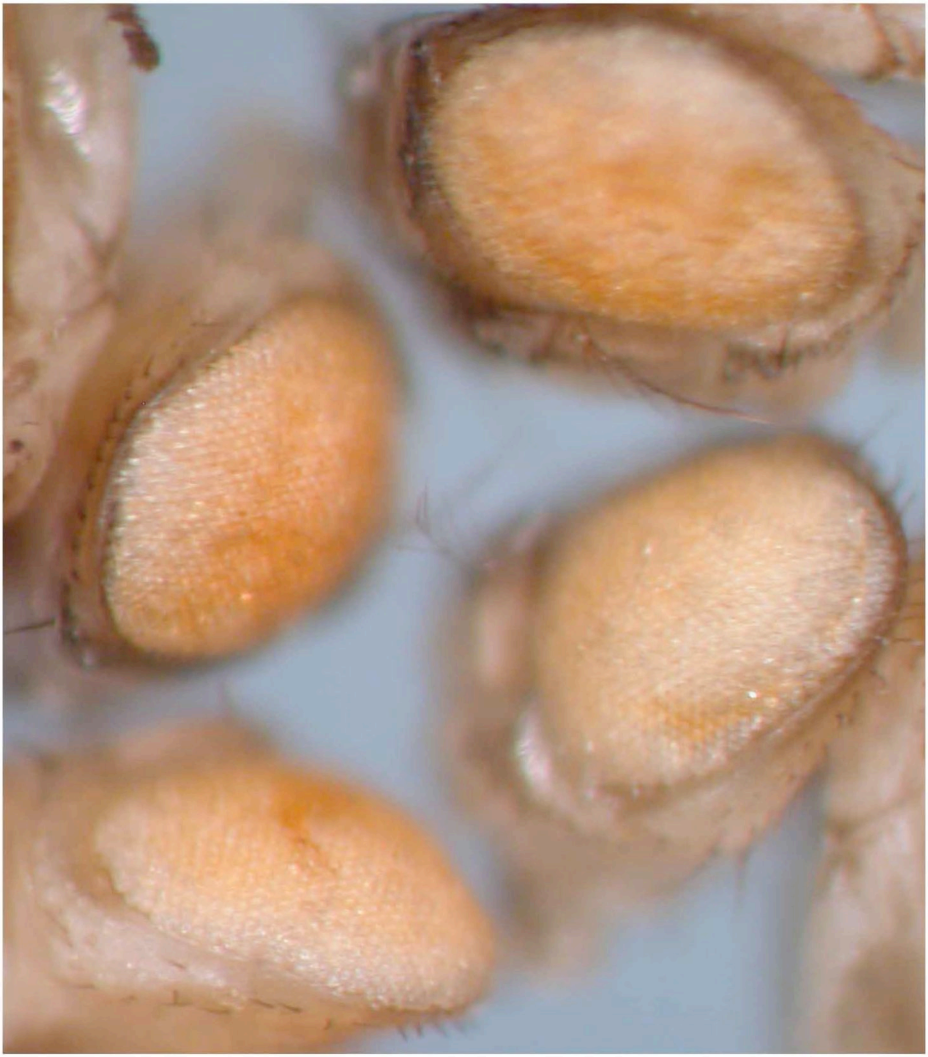

Adh-w/w; w-Adh/w-Adh MKRS/TM3,Ser

Adh-w/w; w-Adh/w-Adh RNA Pol II140(A5)/TM3,Ser
